# Supplementary material for: Determinants of neonatal near misses among neonates admitted to Guji and Borena zones selected public hospitals, Southern Ethiopia, 2021: A facility based unmatched case control study design
Source: PLOS Glob Public Health. 2022 Jan 14;2(1):e0000168. doi: 10.1371/journal.pgph.0000168 (PMC10022009; doi:10.1371/journal.pgph.0000168)
Supplement: S1 Text — (DOCX) [file pgph.0000168.s001.docx]

##

## **INFORMATION SHEET AND CONSENT FORM**

**Title of the Research Project**: “***Determinants of neonatal near misses among neonates admitted to Guji and Borena zones selected public hospitals, Southern Ethiopia, 2021. A facility based unmatched case control study design”***

**Name of Investigators**: Anteneh Fikrie and Elias Amaje

**Name of the Organization**: Bule Hora University

**Introduction**: Good morning /afternoon. My name is Mrs./Mr_____________________________. I am here on behalf of Anteneh Fikrie and Elias Amaje. The information you provide will be crucial in determining the neonatal near miss in Borena and Guji Zones of Public Hospitals. I would be thankful if you spend some time answering questions related to your pregnancy, delivery, life style, neonates and associated factors.

**Purpose of the Research Project**: To identify determinants of neonatal near miss

**Procedure:** after you gave your consent to participate in this research, you will answer certain easy questions and other data’s will be collected from your medical records and observation

**Risk and /or Discomfort:** there is no risk. Because it is after birth interview, clients might get tired. However, the risk of discomfort might not be greater than your contribution to the study.

**Benefits:** contribution to the improvement of the public health because of the information you give is valuable. Can ask anything you want.

**Confidentiality**: Your responses will be kept totally of confidential, no names will be taken

**Person to contact:** This research project was reviewed and approved by the Institutional Review Board of Bule Hora University. If you want to know more information; you can contact the committee through the address below. If you have any question you can contact any of the following individuals (Investigator and Advisors) and you may ask at any time you want.

1. Name of Principal investigator: Anteneh Fikrie(BSC, MPH)

Cell phone: +251-922465129

Email: [antenehfikrie3@gmail.com](mailto:antenehfikrie3@gmail.com)

3. Name of co-investigator: Elias Amaje (BSc, MPH/RH).

Cell phone: +251- 916-64-89-50

E-mail: eliasamaje@gmail.com

May I get your permission to continue gathering data from different records that you have?

1. Yes B) No

Name Responsibility Signature

## **ENGLISH VERSION QUESTIONNAIRES**

| Questionnaire number __________________ Date of interview______________________ | | | | | | | | | | | | | | | |
| --- | --- | --- | --- | --- | --- | --- | --- | --- | --- | --- | --- | --- | --- | --- | --- |
| Name of interviewer_____________________ Name of Supervisor___________________ | | | | | | | | | | | | | | | |
| Name of Hospital ______________________________________Code_______________  Please put “X” or “√” symbol on the blank space Case________ Control ______ | | | | | | | | | | | | | | | |
| **SECTION I: SOCIO-DEMOGRAPHIC AND ECONOMIC DATA** | | | | | | | | | | | | | | | |
| S.No | Question | Option | | | | | | | | Skip | | Code | | | |
|  | Age in full years? | ---------------- years | | | | | | | |  | |  | | | |
|  | Marital status | 1. Single 2. Married 3.Divorced 4.Widowed | | | | | | | |  | |  | | | |
|  | Place of residence? | 1. Urban 2.Rural | | | | | | | |  | |  | | | |
|  | Maternal Educational status | 1. No Education 2. Primary Completed (grade 1-8) 3. Secondary Completed (grade 9-12) 4. College and above | | | | | | | |  | |  | | | |
|  | Paternal Educational status | 1. No Education 2. Primary Completed (grade 1-8) 3. Secondary Completed (grade 9-12) 4. College and above | | | | | | | |  | |  | | | |
|  | Maternal occupational status | 1. Farmer 2. Housewife  3. Government employee 4. Merchant  5.NGO employee 6.Daily laborer  7.Others,specify__ | | | | | | | |  | |  | | | |
|  | Paternal occupational status | 1. Farmer 2. Housewife  3. Government employee 4. Merchant  5.NGO employee 6.Daily laborer  7.Others,specify__ | | | | | | | |  | |  | | | |
|  | Average monthly income | --------------- birr | | | | | | | |  | |  | | | |
| **SECTION II: OBSTETRICS HISTORY OF THE MOTHER** | | | | | | | | | | | | | | | |
|  | What type of pregnancy is it? | | | | Planned 2. Unplanned | | | | |  | | |  | | |
|  | Number of pregnancy including the current? (Gravidity) | | | | ------------- | | | | |  | | |  | | |
|  | How many of your pregnancy were >28 weeks**?(Parity)** | | | | ------------- | | | | |  | | |  | | |
|  | Have you ever had ***an abortion***? (<28 weeks) if yes how many? | | | | 1.Yes ----------- 2.No | | | | |  | | |  | | |
|  | Did you lost a baby within 28 days afterbirth? ***[Previous history of neonatal death, If any]*** | | | | Yes 2. No | | | | |  | | |  | | |
|  | What is the period between last pregnancy and current pregnancy in months***?[ Birth interval]*** | | | | ---------months | | | | |  | | |  | | |
|  | What is the number of your children? | | | | _____________ | | | | |  | | |  | | |
|  | Do you have ANC visit for the current pregnancy? | | | | 1.Yes 2. No | | | | | If no-11 | | |  | | |
|  | How many months pregnant were you at first visit of ANC for the current pregnancy? | | | | ------------months | | | | |  | | |  | | |
|  | How many ANC visits did you have for the current pregnancy***?[Number of ANC visits]*** | | | | -----------times | | | | |  | | |  | | |
|  | How many months pregnant were you when you gave birth of during the current pregnancy**?[Gestational age at birth]** | | | | -------------------months ***(Observe the maternal card]*** | | | | | | | | | | |
|  | Did you experienced water broke ***(Rupture of membrane)*** for the current pregnancy at your home? **[Observe the maternal card record]** | | | | 1.Yes 2. No | | | | |  | | |  | | |
|  | Do the women referred from other health facility? | | | | 1.Yes 2. No | | | | |  | | |  | | |
|  | What is the mode of delivery for the current pregnancy? | | | | 1. SVD 2. CS 3.Instrumental delivery | | | | | | | | | | |
| **SECTION III: MATERNAL MEDICAL HISTORY** | | | | | | | | | | | | | | | |
|  | Did you have diagnosed with anemia? | | | | | | 1.Yes 2. No | | | |  | | | |  |
|  | Did you diagnosed with Hypertension before the current pregnancy? | | | | | | Yes 2. No | | | |  | | | |  |
|  | Did you develop Pregnancy induced Hypertension in this pregnancy? | | | | | | Yes 2. No | | | |  | | | |  |
|  | Did you have Diabetic Mellitus before the current pregnancy? | | | | | | Yes 2. No | | | |  | | | |  |
|  | Did you develop Gestational Diabetic Mellitus in this pregnancy? | | | | | | Yes 2. No | | | |  | | | |  |
|  | Did you diagnose with Syphilis after getting this pregnancy? | | | | | | Yes 2. No | | | |  | | | |  |
|  | Have you ever diagnosed with HIV/AIDS? | | | | | | Yes 2. No | | | |  | | | |  |
|  | What is the weight of the mother? [observe from the record] | | | | | | ________kg | | | | | | | | |
|  | What is the Height of the mother? [observe from the record] | | | | | | ________m | | | | | | | | |
|  | What is the MUAC of the mother? | | | ________cm [observe from the record | | | | | | | | | | | |
|  | Did the mother sustained any one of the following pregnancy complications during the current pregnancy (Tick all the potentials presence)**[Observe from the record]** | | **Complications** | | | | | **Options** | | |  | | |  | |
|  |  |  | Severe APH | | | | | 1.Yes | 2. No | |  |  |  |  |  |
|  |  |  | Severe-preeclampsia | | | | | 1.Yes | 2. No | |  |  |  |  |  |
|  |  |  | Eclampsia | | | | | 1.Yes | 2. No | |  |  |  |  |  |
|  |  |  | Uterine rupture | | | | | 1.Yes | 2. No | |  |  |  |  |  |
|  |  |  | Sepsis | | | | | 1.Yes | 2. No | |  |  |  |  |  |
| **SECTION IV: NEONATAL CHARACTERISTICS** | | | | | | | | | | | | | | | |
|  | Gestational age of neonate at birth | | | | | ----------weeks | | | | | **The interviewer will check the health records of the neonate**  . | | | | |
|  | Neonates gender | | | | | 1. Male 2. Female | | | | |  |  |  |  |  |
|  | Neonates birth weight ( in grams) | | | | | -------------grams | | | | |  |  |  |  |  |
|  | What is Apgar score at 5^th^ minute | | | | | __________ | | | | |  |  |  |  |  |
|  | Did the neonate have respiratory distress/apnea | | | | | 1.Yes 2. No | | | | |  |  |  |  |  |
|  | Blood transfusion | | | | | 1.Yes 2. No | | | | |  |  |  |  |  |
|  | Presence of infection | | | | | 1.Yes 2. No | | | | |  |  |  |  |  |
|  | Persisting signs of respiratory distress for more than one hour. | | | | | 1.Yes 2. No | | | | |  |  |  |  |  |
|  | Requirement for intermittent positive pressure ventilation. | | | | | 1.Yes 2. No | | | | |  |  |  |  |  |
|  | Bile stained vomiting (signs suggesting bowel obstruction) | | | | | 1.Yes 2. No | | | | |  |  |  |  |  |
|  | Feeding problems severe enough to cause clinical concern | | | | | 1.Yes 2. No | | | | |  |  |  |  |  |
|  | Cardiovascular Problems requiring monitoring or intervention | | | | | 1.Yes 2. No | | | | |  |  |  |  |  |
|  | Congenital Malformations that may require intervention | | | | | 1.Yes 2. No | | | | |  |  |  |  |  |
|  | Congenital Malformations that may require intervention convulsion | | | | | 1.Yes 2. No | | | | |  |  |  |  |  |
|  | Surgery | | | | | 1.Yes 2. No | | | | |  |  |  |  |  |
|  | Phototherapy in 24 hours of life. | | | | | 1.Yes 2. No | | | | |  |  |  |  |  |
|  | Parenteral intravenous drugs or nutrition | | | | | 1.Yes 2. No | | | | |  |  |  |  |  |
|  | Any intubation used | | | | | 1.Yes 2. No | | | | |  |  |  |  |  |
|  | If one of the above criteria’s present, classify as **NEONATAL NEAR MISS** | | | | | | | | | | | | | | |
|  | If there is no problem identified among the list of criteria’s classify as **NORMAL NEONATE** | | | | | | | | | | | | | | |
