# Supplementary material for: Determinants of neonatal near misses among neonates admitted to Guji and Borena zones selected public hospitals, Southern Ethiopia, 2021: A facility based unmatched case control study design
Source: PLOS Glob Public Health. 2022 Jan 14;2(1):e0000168. doi: 10.1371/journal.pgph.0000168 (PMC10022009; doi:10.1371/journal.pgph.0000168)
Supplement: Attachment — Submitted filename: 1. Responses to the reviewers.docx [file pgph.0000168.s002.docx]

**Dear reviewer 1**, First of all I would like to thank you very much for your motivating, inspiring and astonishing appreciation towards our manuscript. We are also indebted for your constructive and insightful comments which we believe that it would improve the quality of our manuscript. Thus, according to your comments, and suggestions the following amendments has been taken.

Q1. In the conclusions, the authors cite the use of contraceptives to increase the time between one pregnancy and another.

**Answer:** I have cited the sentences accordingly.

Q2. But, the question remains whether this would be the only or the best alternative to be scored in the conclusions. I believe that it is worth bringing up a little about how public policies are being implemented by the local government, as well as reflecting on the installed capacity of material and human resources to serve this population

**Answer:** Based on your critical comment, I have rewritten the conclusion section in the following manner.

Strong public health policies should be established or needs to be reinforced for the provision of high quality essential health care for mothers, and newborns at the community and health facility levels. The local, regional and national governments should strengthened inter-sectoral collaboration to ensure community empowerment and demand creation for effective use of newborn and child survival interventions with due focus on mothers who had had prior history of adverse pregnancy outcomes and on the poorest and marginalized portions of the rural community requiring equitable distribution of services.

**Dear reviewer 2**, First of all I would like to thank extends my deep indebtedness for your beneficial, insightful and positive comments which i believe that it would improve the quality of the manuscript.

Q1. The authors need to do a thorough edit for the document- there are several spelling and typographical errors.

**Answer:** I did through and in-depth editing in line with your comments.

Q2. Is the abortion miscarriage or induced? Please add the word "prior" before abortion

**Answer**: In this study we considered both miscarriage and induced abortion. I have added the word prior before abortion.

Q3. ---(NMR) to less than 10 per 1000 live births. Is it from the current rate of??

**Answer**: No!! Currently the NMR is 29 per 1000 live births. The government is working aimed to reduce NMR from 29/1,000 live births to less than 10/1,000 live births.

Q4. Sample size calculation is not clear??

**Answer**: Sample size used for case control study is double proportion formula. As a result, for the ease of calculation we used Epi Info™ software which is a trademark of the Centers for Disease Control and Prevention (CDC). The software is in the public domain and freely available for use, copying translation and distribution. Thus, during the sample size estimation we have used the following assumptions proportion of controls exposed 15.8%, proportion of cases with exposure 5.4% from previous studies conducted somewhere , two-sided confidence level 95%, power 80% and ratio of controls over cases 2:1

Q5. ----the face to face structured interview was applied to who? Providers, moms?

**Answer:** ----face-to-face structured interview was applied to the mothers.

Q6.There needs to be a section on **data quality issues** and what is lacking. Is the sample size sufficient, etc.? The authors should also add a few sentences on policy relevance and need for further investigation.

**Answer**: I have incorporated each comment in the sections where they are applicable**.** The data quality issues has been discussed at the methodology section particularly under a sub-section of **“Data collection tools techniques and quality assurance”**

Q7. This association needs a better explanation. Firstly- clarify is you are speaking about miscarriages or induced abortion. Secondly, provide more data from elsewhere to nest your argument

**Answer:** In this study we considered both miscarriage and induced abortion. Likewise, I have encompassed different data after reviewing published articles.

Q8. --------why is this an in-congruency?

**Answer**: It is an editorial problem. It is congruent finding. I have corrected it.

Q9There are many places within the document where I had a question or a suggestion. I have included that as a sticky note in the document. I have also changed text that you will see in a different font.

**Answer**: I have corrected based on your suggestions and comments.
