# Supplementary material for: Determinants of neonatal near misses among neonates admitted to Guji and Borena zones selected public hospitals, Southern Ethiopia, 2021: A facility based unmatched case control study design
Source: PLOS Glob Public Health. 2022 Jan 14;2(1):e0000168. doi: 10.1371/journal.pgph.0000168 (PMC10022009; doi:10.1371/journal.pgph.0000168)
Supplement: Attachment — Submitted filename: 1. Responses to the reviewers Second revsion.docx [file pgph.0000168.s003.docx]

**Dear reviewer 3**: First of all I would like to thank you very much for your motivating, inspiring and astonishing appreciation towards our manuscript. We are also indebted for your constructive and insightful comments which we believe that it would improve the quality of our manuscript. Thus, according to your comments, and suggestions the following amendments has been taken.

- Globally in 2017, 2.5 million babies died from preventable causes like prematurity, complication during the time of birth, bacterial infections, congenital malformations, and poor quality or no health care given at all.”
  - **Answer:** Thank you very much for this insightful comment. I have cited the missed reference.
- Under data processing and analysis, please elaborate on all the covariates and describe them for better understanding of the model.
  - **Answer:** Thank you again for such constructive suggestions. Based on your suggestion, I have included all the covariates based on the comments.
- Why was access to healthcare not evaluated for statistical analysis?
  - **Answer:** Thank you very much for this question. We considered ANC visit (Yes and no) and Number of ANC visit (1-3 visits and ≥4 visits) for assessing access to healthcare. Thus, during the bivariable regression analysis the p-value for “Number of ANC visit” was >0.25.
- Also, I’ve added comments in the article about several typographical errors which authors need to correct.
  - **Answer:** I have corrected accordingly.
